# Empathy and Occupational Health and Well-Being in Ecuadorian Physicians Working with COVID-19 Patients: A Mixed-Method Study

**DOI:** 10.3390/healthcare11081177

**Published:** 2023-04-19

**Authors:** Estefan Matiz-Moya, Roberto C. Delgado Bolton, Esperanza García-Gómez, Luis Vivanco

**Affiliations:** 1 Hospital of Specialties Eugenio Espejo, Quito 170702, Ecuador; 2 Faculty of Health Sciences, International University of La Rioja, 26006 Logrono, Spain; 3 Platform of Bioethics and Medical Education, Centre for Biomedical Research of La Rioja, 26006 Logrono, Spain; 4 Department of Diagnostic Imaging (Radiology) and Nuclear Medicine, University Hospital San Pedro, 26006 Logrono, Spain; 5 Department of Statistics and Operations Research, University of Jaen, 23071 Jaen, Spain; 6 National Centre of Documentation on Bioethics, Rioja Health Foundation, 26006 Logrono, Spain; 7 Faculty of Health Sciences, European Atlantic University, 39011 Santander, Spain

**Keywords:** somatization, emotional exhaustion, work alienation, empathy, COVID-19, doctor-patient relationship

## Abstract

Approximately one out of ten COVID-19 cases in Ecuador was a physician. It has been reported that this situation has led to a serious detriment of physicians’ health and well-being. This study aimed to (i) identify predictors of emotional exhaustion, somatization, and work alienation in Ecuadorian physicians working with COVID-19 patients and (ii) explore the pandemic impact on doctor–patient relationships and on empathy. In 79 Ecuadorian physicians (45 women) who worked with COVID-19 patients, two separate multiple regression models explained the following: 73% of the variability of emotional exhaustion was based on somatization, work alienation, working sector, and passing through a symptomatic infection (*p* < 0.001), and 56% of the variability of somatization was based on gender and emotional exhaustion (*p* < 0.001), respectively. Furthermore, intention to leave the profession was more frequent among physicians with greater work alienation (*p* = 0.003). On the contrary, more empathic physicians never considered leaving their profession during the COVID-19 pandemic (*p* = 0.03). In physicians’ verbatim, cognitive empathy appeared associated to a positive change in doctor–patient relationships. On the contrary, having an overwhelming emotional empathy appeared associated to a negative change in doctor–patient relationships. These findings characterize differences in how physicians cope while working in the frontline of the pandemic.

## 1. Introduction

### 1.1. Clinical Empathy and Occupational Health and Well-Being of Physicians before the COVID-19

In clinical encounters, the definition of empathy is a group of abilities including communication skills and understanding of the views of the patients, together with their inner experiences and feelings, without getting intensely emotionally involved [1]. Three main aspects are representative of this ability: (i) the capacity of understanding patients’ needs and worries related with their disease and treatment; (ii) the capacity of establishing an adequate communication with the patients, even when a language barrier is visible; and (iii) the desire to help them (altruistic motivation). These elements make clinical empathy a mainly cognitive attribute more than emotional. Such distinction between cognition and emotion (and correspondingly, between “empathy” and “sympathy”) may not seem as important in situations different from clinical work, in which both elements could have a similar importance [2]. However, in a clinical encounter, establishing a relationship with the patient mainly based on a cognitive or an emotional response can lead to different outcomes for patients [3] and for physicians [4]. While cognitive empathy refers to an advanced intellectual process (most likely carried out in the cortical area of the brain) that often involves social perception, analysis of information, and the generation of appropriate responses based on the ability to understand patients’ concerns, an emotional empathy refers primarily to a more primitive brain process (most likely carried out in the midbrain and limbic system), in which the response is consequence of an affective resonance of the emotions perceived by the patient. This distinction has important implications in doctor–patient relationships since joining the patients’ emotions (a main feature of sympathy) can hinder a favorable clinical outcome [5]. Recent studies have demonstrated that more empathetic physicians suffer less burnout than those who are more involved with the high emotional load in their daily work [6,7,8,9,10]. 

Before the COVID-19 pandemic began in 2020, being a physician in a public institution in low-to-middle income countries (LMICs) implied working in harsh working environments usually with scarce or inadequately distributed resources and with work overload. In Latin America, where this situation has being reported in recent studies [4,10,11], cognitive empathy has been demonstrated to play a key role in the prevention of work distress in those physicians. 

### 1.2. Physicians’ Health and Well-Being during COVID-19 in LMICs: The Case of Ecuador

Latin America and the Caribbean (LAC) are among those regions most affected by the COVID-19 pandemic worldwide [12]. The COVID-19 pandemic has strained health systems in the region, and Latin-American physicians were one of the most affected groups [13,14,15]. This was the case of Ecuador, where the Pan-American Health Organization (PAHO) estimated in nearly one million the number of individuals with a confirmed COVID-19 diagnosis by 1 November 2022 [16]. According to the same source, the pandemic lead to more than 35,900 deaths in this South-American country. With a case fatality ratio of 3.6, Ecuador occupied, after Peru, the second position in the ranking of South-American fatality ratio due to COVID-19 [16]. To cope with the pandemic, the Ecuadorian government established containment measures and movement restrictions [17]. However, these actions were insufficient and not adequately applied along the country. Furthermore, with a highly fragmented healthcare system, important inequalities appeared, affecting more vulnerable groups. According to an Informative Note published by the International Labor Organization in 2021, healthcare professionals were the ones who most suffered the impact of the pandemic in Ecuador [17]. In most cases, healthcare professionals had insufficient facilities, work overload, and were poorly trained and informed. A recent study measured its impact on the mental health of Ecuadorian health professionals [18]. According to the authors, anxiety, depression, post-traumatic stress, insomnia, obsessive-compulsive symptoms, somatization, and some emotional disorders such as compassionate fatigue increased among health professionals working in Ecuadorian health institutions. This situation was especially accentuated in physicians, female professionals, and in those who worked with COVID-19 patients. Studies reported in other countries [19,20,21,22,23] have found similar findings to the ones described in Ecuador. 

From all healthcare professionals, those who are in the frontline attending and caring for COVID-19 patients were the ones who experienced the greater emotional overload and the ones more exposed to suffering its impact on their health and well-being at the workplace. This situation may negatively affect their professional performance. Some authors even suggest it as an important cause of physicians’ work abandonment or lack of compassionate care due to the emotional fatigue [22,24]. 

### 1.3. Aims of this Study

In this context, the main objectives of this study were: to characterize factors of influence on the variability of emotional exhaustion, somatization, and work alienation, three indicators of deterioration of physical and psychological occupational well-being, in physicians dedicated to attending COVID-19 patients during the pandemic; and to explore the pandemic impact on doctor–patient relationships and on medical empathy. For the first aim, two research objectives were set: (i) to measure symptoms associated with emotional exhaustion, somatization, and work alienation in a sample of Ecuadorian physicians working with COVID-19 patients; and (ii) to identify factors influencing the self-perception of the above-mentioned symptoms from socio-demographic, working, professional, and COVID-19 exposure variables. For the second aim, another two research objectives were established: (i) to compare the scores on medical empathy according to the above-mentioned variables; and (ii) to explore whether self-perceived changes in doctor–patient relationships are mainly associated with specific features of a cognitive or an emotional empathy. 

## 2. Methods

### 2.1. Participants

A sample of physicians, working in healthcare institutions of the Province of Pichincha (Ecuador) attending COVID-19 patients during the pandemic, were contacted and asked if they were willing to take part in this study. Healthcare workers from different areas as nursing, physicians not focusing their clinical work on COVID-19 patients, and physicians working with COVID-19 patients in other territories different than the Province of Pichincha were excluded from this study.

The study design, which had been authorized by an independent ethical committee (Ref. CEImLAR-PI-252), was performed following the guidelines of the Declaration of Helsinki regarding studies with human subjects. All participants of the study signed a web-based informed consent. The sample was obtained from a database of medical graduates of the Faculty of Medicine at the University of Las Americas, Quito, Ecuador. Participation of respondents was anonymous and voluntary. 

### 2.2. Main Measures

Self-perception of somatization, emotional exhaustion and work alienation were used as main measures of indicators of deterioration of physical and psychological occupational well-being. The Scale of Collateral Effects (SCE) of the Questionnaire of General Labour Well-being was applied as measuring instrument [25]. The SCE is composed by three mini-scales: the scale of exhaustion (SE) with 4 items; the scale of somatization (SS) with 5 items; and the scale of work alienation (SA) with 4 items. Each item of the abovementioned mini-scales starts with the following statement: “*Currently, because of my work, I feel:*” followed by one specific symptom. Symptoms included in the SE are: work overload, emotional exhaustion, physical exhaustion, and mental saturation. The SS measures the following symptoms: digestive disorder, back pain, insomnia, headache, and muscle tension. Finally, the SA evaluates: bad mood, low personal fulfilment, depersonalized treatment, and frustration. The perception of each symptom is answered following a 7-point Likert-type scale reflecting a daily frequency in the last week from 1 (never) to 7 (always). The original version of the SCE was designed in Spanish language. This instrument has been initially tested in professionals from different disciplines in Spain and in Latin American countries, including Ecuador, showing good psychometric properties [25]. In physicians, this instrument has been previously proven with samples from Spain [7] and Latin American [4,10,11,26] countries with excellent results.

In addition, the Health Professional version of the Jefferson Scale of empathy (JSE-HP) [27] was used for measuring medical empathy. The JSE-HP (20 items) is answered in a 7-point Likert scale from 1 (strongly disagree) to 7 (strongly agree). Higher scores in the JSE-HP are associated with greater self-perception of empathic abilities. The Spanish version of the JSE-HP, initially tested and validated with Spanish and Latin American healthcare professionals, including physicians, has demonstrated consistent psychometric properties [27].

Questionnaires were accompanied with a form including socio-demographic variables (age, gender, civil status, cohabitation, family burden), professional and working variables (years of professional experience, medical specialty, working sector), and exposure to COVID-19 variables (having symptoms compatible with COVID-19, persistent COVID-19, cohabitants with COVID-19 symptoms, and cohabitants deceased due to COVID-19). Finally, information related to their doctor–patient relationship (DPR) during the pandemic was collected in a semi-structured form. In this form, respondents indicated: (i) the main DPR model they established in their daily work from the four proposed by Ezequiel and Linda Emanuel [28]; if, during the pandemic, they had the intention to: (ii) leave their job or (iii) their medical profession; and (iv) if they considered that their DPR improved, maintained, or eroded after the pandemic. Those who answered that they perceived a change (negative or positive) in their DPR were asked to describe the main changes that occurred.

### 2.3. Analysis

Only psychometric instruments with alpha coefficients equal to or higher than 0.70 were included in the analyses. Emotional exhaustion, somatization, work alienation, and empathy were used as dependent variables. After normality was assessed, using Pearson’s chi-squared and Lilliefors–Kolmogorov–Smirnov tests, Spearman’s correlation analyses were performed in order to determine statistical associations among emotional exhaustion, somatization, work alienation, empathy, age, and years of professional experience.

For categorical variables, comparative analyses using non-parametric Mann–Whitney U-tests were completed. Effect size (*r*) was calculated applying the formula published by Fritz, Morris and Richler [29] and Tomczak and Tomczak [30] for non-parametric tests. Taking into consideration indications from Hojat and Xu [31], an *r*-value equal to 0.50 was categorized as a large effect size with a key practical importance; equal to 0.30 was categorized as a medium effect size with a moderate practical importance; and equal to 0.10 was categorized as a small effect size with a negligible practical importance. Finally, separate multiple linear regression analyses were completed using emotional exhaustion, somatization, and work alienation as dependent variables, while all the other variables with statistical significance in correlation and comparative analyses were used as potential predictors. A regression model was accepted only if conditions of statistical inference (normality, zero mean, constant variance and uncorrelatedness of the residuals, in addition to linearity and absence of multi-collinearity) were met. Effect sizes (Cohen’s-*f*^2^) were calculated by each model obtained. A value equal to or greater than 0.02 and smaller than 0.15 was interpreted as a small effect, equal to or greater than 0.15 and smaller than 0.35 as a medium effect, and equal to or greater than 0.35 as a large effect, following the interpretation criteria proposed by Cohen [32]. 

All analyses were performed using R statistical software, version 4.1.1., for Windows. The statistical analyses of the data also included *multilevel* [33], *rstatix* [34], *lsr* [35], and *nortest* [36] packages.

Verbatim collected in the semi-structural forms were grouped based on the presence/absence of the term empathy, or specific features of empathy and sympathy [1], such as: communication of understanding, state of mind (intellectual vs. emotional), behavioral motivation (altruistic vs. egoistic), or key mental-processing mechanism (cognitive/intellectual/understanding vs. affective/emotional/feeling). 

## 3. Results

From the 104 physicians who initially accepted to participate in the study, 79 (45 women) returned fully answered surveys and were included into the analysis. The average age of this sample was 35 (*SD* = 10) years old, ranging between 24 and 66 years old. The average professional experience in the entire sample was 7 years (*SD* = 8), with a range between 1 and 30 years of medical practice. By specialty, 30 (43.5%) did not have a medical specialty (general practitioners), while the other 49 (56.5%) were distributed in 14 specialties including family medicine, emergency medicine, internal medicine, pneumology, anesthesiology, oncology, or geriatrics. The summary of the descriptive analysis by all variables collected is shown in Table 1. 

Regarding the scales used, all of them showed adequate psychometric properties with values similar to the ones reported in previous studies with Latin American physicians [4,10,11,26,27]. The score distribution, descriptive statistics, and reliability of the instruments used are described in Table 2.

With regard to the first aim related to the characterization of factors of influence in the variability of indicators of physical and psychological occupational well-being, three separate analyses were performed for somatization, exhaustion, and alienation, respectively.

**Figure 1:**
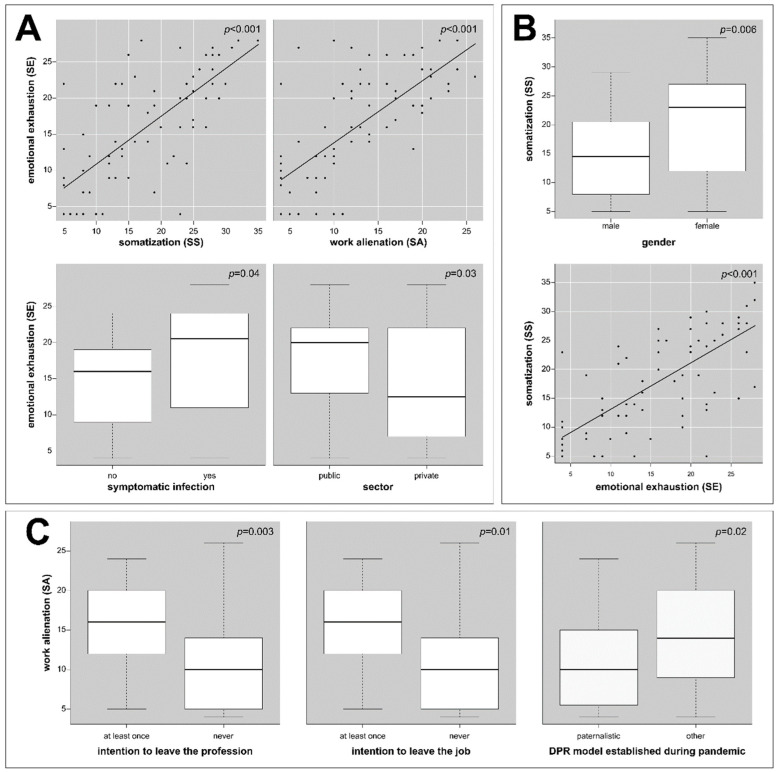
Scores of emotional exhaustion (**A**), somatization (**B**), and work alienation (**C**) by variables with statistical significance in Ecuadorian physicians who worked with COVID-19 patients. SE, scale of exhaustion; SS, scale of somatization; SA, scale of alienation.

In the case of emotional exhaustion, a positive correlation with somatization (ρ = +0.73; *p* < 0.001) and with work alienation (ρ = +0.72; *p* < 0.001) was confirmed. Neither empathy (ρ = −0.20; *p* = 0.08) or age (ρ = +0.23; *p* = 0.05) variables were correlated with exhaustion. However, both variables were closer to the statistical significance. Finally, no correlation was observed between exhaustion and years of professional experience (ρ = +0.11; *p* = 0.35). Mann–Whitney U-tests confirmed a greater exhaustion in physicians who worked in public institutions (*p* = 0.02; *r* = 0.26), suffered symptomatic COVID-19 infection (*p* = 0.02; *r* = 0.27), had the intention to leave their job (*p* = 0.008; *r* = 0.30), and had the intention to leave their profession (*p* = 0.009; *r* = 0.30). Based on these findings, a regression model was created (Table 3) explaining the 74% of variance in emotional exhaustion (R^2^-adjusted = 0.73; F_(1,71)_ = 50.6; *p* < 0.001) with a large effect size (Cohen-*f*^2^ = 2.85). According to this model, greater somatization (*p* < 0.001), greater alienation (*p* < 0.001), working in a public health institution (*p* = 0.03), and having suffered a symptomatic infection with COVID-19 (*p* = 0.04) appeared as predictors of greater emotional exhaustion in Ecuadorian physicians (Figure 1A).

In the case of somatization, in addition to the previously reported correlation with emotional exhaustion, a negative correlation with empathy (ρ = −0.23; *p* = 0.04), and a positive correlation with work alienation (ρ = +0.58; *p* < 0.001), were observed. Neither age (ρ = +0.21; *p* = 0.07) or years of professional experience (ρ = +0.19; *p* = 0.10) correlated with somatization. Mann–Whitney U-tests showed a greater somatization in female physicians (*p* = 0.007; *r* = 0.31), in physicians who considered leaving their job (*p* < 0.001; *r* = 0.38), and leaving their profession (*p* = 0.005; *r* = 0.32). Taking into account the findings described, a regression model using multiple linear regression analysis was created, as is presented in Table 3. This model explained 58% of the variance in somatization (R^2^-adjusted = 0.56; F_(1.74)_ = 50.2; *p* < 0.001) with a large effect size (Cohen-*f*^2^ = 1.36). According to it, women (*p* = 0.006) and having greater emotional exhaustion (*p* < 0.001) appeared as predictors of greater somatization in Ecuadorian physicians working with COVID-19 patients (Figure 1B). 

Finally, in the case of work alienation, neither empathy (ρ = −0.21; *p* = 0.07), age (ρ = +0.02; *p* = 0.90) nor years of professional experience (ρ = +0.01; *p* = 0.92) showed a correlation with this variable. A Mann–Whitney U-test showed a greater work alienation in physicians who had the intention to leave their profession (*p* = 0.003; *r* = 0.34), and to leave their job (*p* = 0.01; *r* = 0.29), and those who established a doctor–patient relationship different from a paternalistic one during the pandemic (*p* = 0.02; *r* = 0.26). These findings are represented in Figure 1C. The work alienation variable was also analyzed with a multiple regression model. Nevertheless, regression models resulting from this analysis did not comply with all the conditions required for statistical inference.

**Figure 2:**
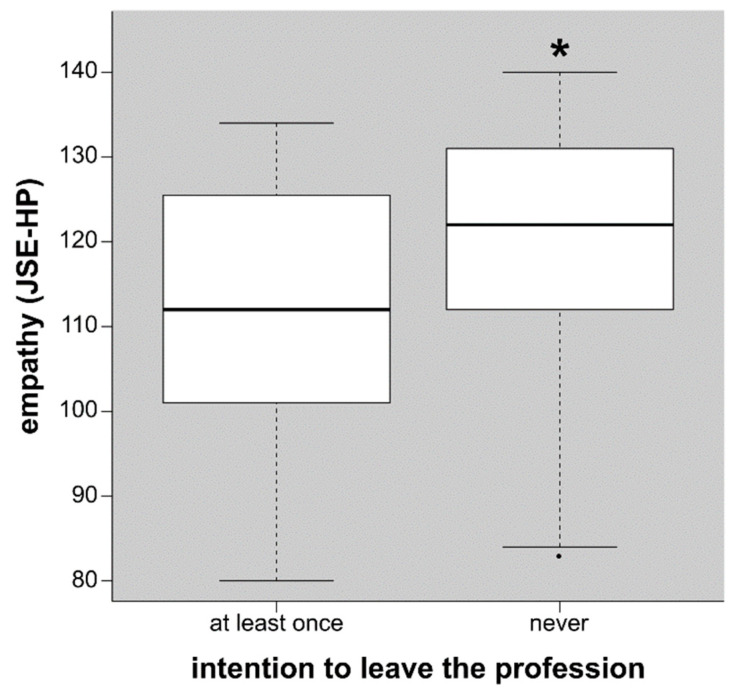
Scores of clinical empathy by the intention to leave the medical profession in Ecuadorian physicians who worked with COVID-19 patients. JSE-HP, Jefferson Scale of Empathy, Healthcare professional version. * *p* < 0.05. Outlier is represented by a dot.

Regarding the second aim, exploring the pandemic impact on doctor–patient relationships and on medical empathy, comparative analyses based on Mann–Whitney U-tests showed differences in empathy only by the variable intention to leave the profession (*p* = 0.03) with a small effect size (*r* = 0.25). This finding is shown in Figure 2. 

From the 24 physicians who reported a change in their doctor–patient relationships and described the main aspect changed, 16 considered this change as positive, while the other eight considered it as negative. In the first group, cognitive empathy (i.e., «I have learnt to be more empathic»), and its components such as, empathic understanding (i.e., «I have learnt to understand their symptoms and I am considerate towards them when proposing a treatment plan. Now, I understand the recuperation is different for each person»), communication of understanding (i.e., «I try to provide the most complete information and help to improve the lifestyle of my patients»), or developing a more altruistic behavior (i.e., «I think each patient must be treated the way we would treat one of our own family»), were mentioned in nine cases. In this group, seven physicians associated this change with others aspects not related to empathy (i.e., «Patients are now coming more informed about their health conditions» or «There are more reliable information sources easy to access»). In the second group, six physicians described two specific features of sympathy—being more egoistic and self-centered (i.e., «During the consultation I am more focused on myself than on the patient») and having a greater affective distance (i.e., «I am more insensitive and even indolent, to the point of limiting the physical exam of the patients»)—as the main negative change in their consultations with the patients. In this group, another two physicians associated this deterioration in their relationships with the patients with aspects not related neither to empathy or sympathy (i.e., «There is much more self-medication by the patients and that makes it more difficult choosing the best treatment»). The summary of the verbatim of all respondents is shown in Table 4.

## 4. Discussion

Taking into account Cronbach’s alpha coefficients equal or higher than 0.70, generally recommended by guidelines, but also by the American Educational Research Association, all instruments showed adequate psychometric properties. [37]. 

In studied sample, punctuations on emotional exhaustion, somatization, and work alienation were a little above those previously reported in two studies in healthcare professionals from Spain [7] as well as Mexican, Ecuadorian, Colombian, and Argentinean [10] public healthcare institutions before the COVID pandemic started. This finding suggests a greater self-perception on the three above indicators of deterioration of physical and psychological occupational well-being measured in the study group, which is in coincidence with similar findings reported in other three studies performed with Ecuadorian healthcare workers during the COVID pandemic [15,17,18]. However, these scores were not as high as the ones recently reported in another study performed in Paraguay [38], which has one of the lowest-scored healthcare indicators of Latin America [39]. In the case of empathy, global scores in the Ecuadorian sample were similar to previously described in physicians from Spain and from other Latin American countries [7,10,27]. 

Regarding the first aim of this study, the findings observed confirm that a greater somatization and a greater work alienation are two main predictors of emotional exhaustion. This is in agreement with results from a different study, also in Latin American healthcare professionals, in which exhaustion, somatization, and work alienation were positively correlated [10]. Furthermore, working in the public sector and suffering a symptomatic infection of COVID-19 appear as another two predictors of a greater emotional exhaustion among Ecuadorian physicians. These findings are also in accordance with other studies performed in Ecuador, where working conditions at public healthcare institutions and suffering a symptomatic COVID infection were reported as important risk factors of psychological distress in healthcare workers [15,18,40]. Additionally, analyses revealed that having a greater emotional exhaustion and being a female physician appeared as the two main predictors of a greater somatization in physicians working with COVID patients. Female healthcare workers were also described as a vulnerable group of suffering mental health problems in another study performed in Ecuador [15]. Finally, the findings reported in this study reveal that differences in work alienation measures are mainly explained by the intention to leave the medical profession, the job, and those who established a DPR model different from a paternalistic one. Definitions of work alienation include “a phenomenon that distances workers from their jobs and causes a feeling of meaninglessness, powerlessness, and self-estrangement” [41]. In consequence, a greater work alienation in physicians with the intention of leaving their job and their profession during the pandemic coincides with others, also reported in physicians and nurses, where the intention of leaving the work position, job, or even the profession is connected with a greater job dissatisfaction, burnout, and lack of motivation at the workplace [42,43,44]. According to Emanuel and Emanuel [28], the paternalistic model, when focused on the physician–patient interaction, underlines the requisite that medical interventions on the patient should always be aimed at promoting health and well-being in the best possible way. The premise upon which this model is based is that physicians apply knowledge and clinical skills to evaluate the patients’ medical condition and to detect the treatments that will restore their health or ameliorate their pain with the highest probability. In this model, the physician should be the patient’s guardian, providing what is best for the patient [45,46]. It is generally accepted that this model is justified in emergencies (such as the one experienced during the COVID pandemic, especially in the most critical moments) when time needed to obtain informed consent could irreversibly harm the patient [28,46,47,48]. Consequently, it is plausible that those physicians who were not aware of the legitimacy of this model under such clinical circumstances and tried to establish other DPR models (such as deliberative, informative, or interpretative ones) suffer a greater meaninglessness, powerlessness, or self-estrangement in their daily work.

Regarding the second aim of this study, the findings reported in this study confirm that less empathetic physicians are in higher risk of leaving their profession or at least of losing their professional motivation. This finding brings new experimental evidence supporting the important role that clinical empathy, as a core component of professionalism in medicine, plays in this matter [1]. Furthermore, in accordance with previous studies demonstrating the positive impact that empathy has in the early development of other specific components of medical professionalism [4,49], this finding confirms that empathy offers an important resource to cope with adverse working conditions such as those experienced during the COVID pandemic. In this regard, this finding provides new evidence supporting the role that empathy plays in the prevention of work distress and burnout in physicians [6,8,50]. One of the reasons that can explain this role is in its nature. Empathy, in the specific context of clinical encounters, has been defined as a predominantly cognitive (rather than affective or emotional) ability characterized by three specific features: understanding (of patients’ experiences and concerns), good and clear communication, and altruistic motivation (expressed in a compassionate attitude to care a person in need). An empathetic engagement based on the three above-mentioned features not only protects from negative effects derived from an intensive emotional involvement (as the one experienced by physicians working with COVID patients in their daily work journeys) but also creates a positive and more satisfactory working environment even in adverse working circumstances. This effect has been proven in previous studies with healthcare professionals [4,10,11]. Furthermore, the textual analysis of verbatim collected in this study corroborates the different role that a cognitive or an emotional empathy played in physicians who worked with COVID patients. While specific features related to a cognitive empathy were associated with a positive change in doctor–patient relationships, specific features related to an emotional empathy were associated with a negative change in doctors’ relationships with their patients. These findings corroborate the distinction established by some medical educators regarding the conceptual frame of clinical empathy, and they bring novel evidence supporting the “invert U shape” effect that emotional empathy has not only in clinics but also in physicians’ health and well-being. This inverted U shape has being described by Hojat as follows [1], (pp. 80–81) “the relationship between empathy and positive clinical outcomes is linear (that is, the outcomes progressively become better as a function of an increase in empathic engagement), and the relationship between sympathy and clinical outcomes resembles an inverted U shape (similar to that between anxiety and performance) are confirmed”. In other words, although an excess of empathy is always positive, too much sympathy (emotional empathy) is detrimental. This idea, initially proposed in the context of clinical outcomes, can be also applied in the context of physicians’ occupational health and well-being according to the verbatim collected in this study. In summary, the results of this study show that in adverse working environments (such as the one experimented during the pandemic), a greater development on cognitive empathy abilities offers personal resources that are necessary to prevent emotional overload and to maintain an adequate working performance in clinical encounters with the patients.

*Limitations.* The study included a heterogeneous group of physicians working in two very different environments: public and private institutions. In addition, not all questionnaires were fully answered, and the sample corresponded to one specific territory. However, taking into account that the province where this study was performed was one of the two provinces more affected by the pandemic, the authors considered that this limitation was not crucial, and the study sample reflected important aspects of the experiences lived. 

Another aspect is related to the fact that this study was conducted only with physicians, while other healthcare professionals (such as nurses) were not included. Therefore, its generalizability to other healthcare disciplines different from medicine is limited. However, the authors agree that even if there are some similarities, other important aspects related to the type of work make it necessary to perform a different study specifically focused on nurses and on other healthcare disciplines different from medicine.

## 5. Conclusions

The results underline the key role of empathy in physicians’ health and welfare and the different roles that cognitive and emotional empathy played during the pandemic. Moreover, the results of this study offer new clues of elements that play as risk factors of greater emotional exhaustion, somatization, and work alienation in physicians who were working with COVID patients. 

These results also indicate the urgent need to introduce relevant modifications in the organizational culture of healthcare institutions and in the importance of enhancing empathic abilities from the early stages of the medical career with the objective of decreasing the negative effect that work overload has on the health and well-being of physicians.

Future lines of research could focus on follow-up studies to confirm the findings reported in this study. Based on the above-mentioned findings, interventional studies focused on the reinforcement and development of emotional regulation, communication abilities, and key mental-processing mechanisms (such as understanding patients’ needs and concerns) could offer a valuable tool not only in the acquisition of greater empathy with the patients but also in the reduction in burnout. In addition, further observational studies with bigger samples could also provide more evidence for clarifying the exact role that clinical empathy plays in this matter, either as a direct factor of influence in the reduction in work distress or as a moderator or mediator of other variables of influence directly involved. Furthermore, the findings reported in this study highlight the urgent need to introduce important changes in the organizational culture of healthcare institutions, especially in those that carried intense clinical work during the pandemic. One area appears as a potential target in future studies: the measurement of the positive impact that the reinforcement of services centered on psychological and occupational support has in the occupational well-being of healthcare professionals more exposed to emotional exhaustion. In this matter, developing targeted training programs focused on the acquisition and enhancement of cognitive aspects related to clinical empathy appears to be a valuable alternative. 

## Figures and Tables

**Table 1:** Descriptive analysis of socio-demographic, professional, and working variables, and variables associated with the exposure to COVID-19 (*n* = 79).

Variable	*n* (%)
*Socio-demographic variables*	
Gender	
Male	32 (41.6)
Female	45 (58.4)
Civil status	
Single	46 (59.7)
Married	31 (40.3)
Cohabitants during pandemic	
Without cohabitants	16 (20.8)
With parents	26 (33.8)
With couple	35 (45.4)
Family burden	
No	46 (59.7)
Yes	31 (40.3)
*Professional and working variables*	
Specialty	
Without specialty (general practitioner)	30 (43.5)
With specialty	49 (56.5)
Working sector	
Public	46 (60.5)
Private	30 (39.5)
*Exposure to the COVID-19*	
The respondent has suffered at least one symptomatic infection due to COVID-19	
No	29 (37.7)
Yes	48 (62.3)
The respondent has suffered symptoms compatible with “persistent COVID-19” disease	
No	44 (64.7)
Yes	24 (35.3)
The respondent had a cohabitant with a symptomatic infection due to COVID-19	
No	45 (60)
Yes	30 (40)
The respondent had a cohabitant who died during COVID-19 pandemic	
No	67 (89.3)
Yes	8 (10.7)
*Impact of COVID-19 pandemic in respondent’s daily work*	
“My daily working relationship with the patients during the pandemic was mainly:”	
Paternalistic	35 (46)
Other	41 (54)
“I perceived a change in my relationship with the patients after the pandemic”	
Yes, it is worst	8 (10.7)
No, it is the same	48 (64)
Yes, it improves	19 (25.3)
Having the intention to leave the job during the pandemic	
Never	36 (46.7)
At least once	41 (53.3)
Having the intention to leave the medical profession during the pandemic	
Never	46 (59.7)
At least once	31 (40.3)

**Table 2:** Descriptive analysis and reliability coefficients (*n* = 79).

Statistics	SE	SS	SA	JSE-HP
Range possible	4–28	5–35	4–28	20–140
Range observed	4–28	5–35	4–26	80–140
Mean	16	18	13	116
Standard deviation (*SD*)	8	8	6	15
Quartile				
1st	10	11	8	107
2nd (Median)	17	18	12	119
3rd	22	25	19	128
Reliability (Cronbach alpha)	0.94	0.89	0.86	0.85

JSE-HP, Jefferson Scale of Empathy; SS, Scale of somatization; SE, Scale of exhaustion; SA, Scale of work alienation.

**Table 3:** Multiple linear regression models for somatization and emotional exhaustion measures.

Dependent Variable	Predictors	*β*	*SE*	*t*	*p*
Emotional exhaustion (SE)	Somatization (SS)	+0.40	0.07	+5.88	<0.001
	Work alienation (SA)	+0.54	0.09	+6.32	<0.001
	Sector (private)	−2.18	0.95	−2.29	0.03
	Symptomatic infection (yes)	+2.05	0.97	+2.11	0.04
Somatization (SS)	Gender (female)	+3.67	1.30	+2.82	0.006
	Emotional exhaustion (SE)	+0.77	0.08	+9.08	<0.001

SS, scale of somatization; SE, scale of exhaustion; *β*, beta coefficient; SE, standard error; *t*, *t*-value; *p*, *p*-value.

**Table 4:** Verbatim of changes self-perceived by physicians in their relationships with the patients.

Type of Change	Respondent: «Verbatim»	LE/S (*n*)
Negative	«I see myself more distant and less involved with the patients» «During the consultation I am more focused on myself than on the patient» «I am more suspicious and I avoid physical contact» «I limit physical examination of the patients and dedicate less time in consultations» «I have limited the physical contact with the patients» «I am more insensitive and even indolent, to the point of limiting the physical exam of the patients»	Yes (6)
	«There is much more self-medication by the patients and that makes it more difficult choosing the best treatment» «More protocols and they make more difficult for patients to access their appointments»	No (2)
Positive	«I have improved asepsis and empathy with my patients» «I have learnt to be more empathic» «I feel more empathy for my patients» «I try to provide the most complete information and help to improve the lifestyle of my patients. To do this, I have learnt to be more empathic with them [patients] and more aware of their personal and economic situation, a part of their physical and mental health» «With the pandemic, I have learnt that we are frail and that in any moment we can cease existing. I have learnt to be more empathic and give emotional support to my patients and their families. I think each patient must be treated the way we would treat one of our own family» «I am more sensitive to the needs of my patients» «I have learnt to be more empathic and give emotional support to my patients and their families» «I have more empathy and care towards the more vulnerable people» «I have learnt to understand their symptoms and I am considerate towards them when proposing a treatment plan. Now, I understand the recuperation is different for each person»	Yes (9)
	«Now I put more attention to the time dedicated to washing my hands and using the mask» «I have incremented the personal hygiene and the preventive measures for respiratory tract diseases» «I take care of asepsis» «The people are more conscious now of their surrounding and that is good» «There are several symptoms and signs of this disease that are pathognomonic and others no» «Patients are now coming more informed about their health conditions» «There are more reliable information sources easy to access»	No (7)

LE/S, Linked to empathy/sympathy or some its components.

## Data Availability

Data are available upon request.
